# Successful use of recombinant activated factor VII for postoperative associated haemorrhage: a case report

**DOI:** 10.1186/1757-1626-1-361

**Published:** 2008-11-29

**Authors:** Konstantinos Vlachos, Fotis Archontovasilis, Artemisia Papadima, Dimitrios Maragiannis, Stavros Aloizos, Emmanuel Lagoudianakis, Ioannis G Dalianoudis, Nikolaos Koronakis, John Chrysikos, Spyros Zaravinos, Andreas Manouras

**Affiliations:** 1 1^st ^Department of Internal Medicine, 401 General Army Hospital of Athens, Athens, Greece; 2 Intensive Care Unit, 401 General Army Hospital of Athens, Athens, Greece; 3 1^st ^Department of Propedeutic Surgery, Hippokrateio General Hospital, University of Athens, Athens, Greece; 4 1^st ^Surgical Department, 401 General Army Hospital of Athens, Athens, Greece; 5 2^st ^Department of Surgery, 417 NIMTS, Athens, Greece

## Abstract

**Background:**

Coagulopathy is a major contributing factor to bleeding related mortality even after achieving adequate surgical control of the haemorrhage in trauma and surgical patients.

**Case presentation:**

A 65 years old Greek man was admitted in our ICU with critical haemorrhage following renal biopsy. Despite surgical exploration the patient continued to bleed resulting in a vicious cycle of transfusion, coagulopathy and re-bleeding. After all standard management options were exhausted, the patient was given rFVIIa (total dose 4,8 mg). Clinical improvement was noted without adverse thrombotic complications. One month later the same patient was operated on for a suspected retroperitoneal infected collection that it was assumed to be the cause of persistent pyrexia. After abdominal washout, he suffered haemorrhagic shock with postoperative coagulopathy. Standard transfusion therapy was again unsuccessful. The patient was given rFVIIa again resulting in an immediate reduction in coagulopathic haemorrhage accompanied by a significant improvement in laboratory measurements and reduction in blood products requirements.

**Conclusion:**

Published clinical experiences for the use of rFVIIa in trauma patients are limited to small series and case reports. However, in trauma patients, administration of rFVIIa appears to be effective in addition to prompt surgical intervention as an adjunctive haemostatic measure to control life threatening bleeding in appropriately selected patients.

## Background

Coagulopathy is a major contributing factor to bleeding related mortality even after achieving adequate surgical control of the haemorrhage in trauma and surgical patients, particularly when associated with metabolic acidosis and hypothermia [1,2].

Recombinant activated factor VII(rFVIIa) has been approved since nearly a decade for the prevention and treatment of bleeding episodes in haemophilic patients with inhibitors to coagulation factor VII or factor IX [3]. Recent studies reports the successful management of massive obstetric haemorrhage with the use of rFVIIa[4]. However, administration of rFVIIa appears to be effective in addition to prompt surgical intervention as an adjunctive haemostatic measure to control life threatening bleeding in trauma patients as well as postoperative haemorrhage. Furthermore, recent case reports [5-7] and case series II [1,8-12], have also suggested a role of rFVIIa in the management of life threatening bleeding in patients with trauma induced coagulopathies who do not respond to conventional treatments.

We describe the successful use of rFVIIa in the management of a patient with no pre-existing coagulopathy who developed refractory bleeding following renal biopsy, for persistent proteinuria (2,5 gr/day), despite adequate therapy.

## Case presentation

A 65 year old Greek man with unremarkable previous medical history, was admitted to our ICU because of haemorrhage after renal biopsy. On admission, examination revealed a respiratory rate of 30/min, a pulse rate of 138/min and a blood pressure of 79/58 mmHg. He was in a critical condition with profound hypovolaemic shock, acute renal failure and coagulopathy. Despite volume resuscitation and transfusion of 8 units of Red Blood Cells (RBC) and 4 units of Fresh Frozen Plasma (FFP) the patient developed a rapidly expanding abdomen. He was transported immediately to the operating room for laparotomy.

His preoperative airway examination was unremarkable: mouth opening, oropharyngeal view, thyromental distance, and neck movement were all normal. For anaesthetic induction, after three minutes of pre-oxygenation with 100% O_2 _and initiation of cricoid pressure, the patient received midazolam 2 mg iv, fentanyl 100 mcg iv, etomidate 16 mg iv, and succinylcholine 80 mg iv.

Anaesthesia was maintained with sevoflurane (MAC 1–1.2) in air and oxygen (FiO_2 _50%), and intermittent boluses of fentanyl and rocuronium. The standard monitoring included ECG, capnometry, invasive measurement of blood pressure and central venous pressure, pulsoximetry, diuresis, esophageal and tympanic temperature. Bispectral analysis of the electroencephalogram was used as a guide for anaesthetic depth and bispectral index levels were maintained between 40 and 55. Ligation of the bleeding left kidney vessels was accomplished.

After completion of the operation mechanical ventilation was continued in ICU. Four hours later the patient became haemodynamically unstable with a rapid drop of his haematocrit, while he required increased doses of fluids and vasopressors. A 4-lumen central venous catheter and a pulmonary artery catheter were inserted via puncture of the internal jugular vein. In addition, 4 units of RBC, 13 units of FFP and 6 units of platelets were administered. His status did not stabilise and he was taken directly to the operating room where bleeding from splenic vessels was found. The same philosophy of anaesthetic management is being applied to the perioperative period. Emergency splenectomy was carried out. As his condition further deteriorated administration of rFVIIa, as last effort to control his bleeding, was decided. A total dose of 4,8 mg (60 μg/kg) of rFVIIa along with concurrent transfusion of 4 units of FFP, 2 units of RBC and 6 units of platelets were given. Remarkably, all signs of bleeding appeared to cease (Table 1), the patient's haemodynamic parameters also stabilised and his tissue perfusion and metabolic acidosis gradually improved. There were no further episodes of recurrent haemorrhage. On the second postoperative day, his trachea was extubated in the operating room and thereafter he was discharged home on postoperative day 6.

**Table 1:** Compering charts of haemodynamic parameters after the use of rFVIIa for the 1^st ^time

	**Before the use of rFVIIa (4,8 mg)**	**After the use of rFVIIa (4,8 mg)**
**PT**	24,1	14,1

**aPTT**	54,7	47,7

**INR**	2,15	1,24

PT: Prothrombin Time

aPTT: Partial Prothrombin Time

INR: International Normalisation Ratio

One month later the patient was operated on for a suspected retroperitoneal infected collection that it was assumed to be the cause of persistent pyrexia. After abdominal washout he suffered another episode of severe intraabdominal bleeding with postoperative coagulopathy. Despite the transfusion of 30 units of FFP, 18 units of RBC and 6 units of platelets to control his bleeding, no substantial benefit was observed. We, thereafter, decided to administer again 12 mg (150 μg/kg) of rFVIIa within 16 hours, which resulted in cessation of bleeding shortly after the last dose of rFVIIa with no recurrent episodes of haemorrhage, and correction of clotting parameters (Table 2).

**Table 2:** Compering charts of clotting parameters after the use of rFVIIa for the 2^nd ^time

	**Before the use of rFVIIa (12 mg)**	**After the use of rFVIIa (12 mg)**
**PT**	16,4	11,7

**aPTT**	148,4	40,7

**INR**	1,44	0,99

PT: Prothrombin Time

aPTT: Partial Prothrombin Time

INR: International Normalisation Ratio

## Discussion

The mechanism of coagulopathy in trauma is complex and multi factorial. It includes dilutional coagulopathy, hypothermia, acidosis, hyperfibrinolysis, anaemia and extensive consumption of platelets and coagulation factors [13,14].

Administration of high doses of rFVIIa results in a huge increase of rFVIIa compared with the physiologic state, leading to faster and higher thrombin generation [9]. The recently developed cell base model of coagulation suggests that rFVIIa enhances haemostasis at the site of injury without systemic hypercoagulable effect [13]. In our report we used doses 4,8 mg (60 μg/kg) and 12 mg (150 μg/kg) of rFVIIa respectively. Because widely different doses have been used in the published reports, the optimal dosing regimen for rFVIIa has not yet been established.

Our observation regarding the apparent efficacy of rFVIIa is consistent with a growing body of evidence suggesting a role of rFVIIa for the management of bleeding in surgical patients [1,4,6,9-11,15]. Complete correction of the INR, shortening of the PT and marked improvement of aPTT was achieved after treatment with rFVIIa in our patient. However, normalisation of laboratory measurements of coagulation, in particular the PT, is rather artificial and does not appear to reflect correction of in vivo coagulation [15].

Previous clinical experience with rFVIIa supports a good safety profile. More than 6500 patients have received rFVIIa and only 17 adverse events have been reported. In the reviewed, 4% thromboembolic events were noted, of which 1% occurred in trauma patients[10]. In another study less that 0,05% of serious thromboembolic events occurred in 400.000 doses[13]. In our patient there was no clinical evidence of thromboembolic events.

Its relative high cost would be offset by the costs that would otherwise occur after additional blood transfusions, surgical interventions, shock and other adverse events associated with haemorrhage. We should also consider the benefits of reduced immunologic and viral load, and the decreased implication of organ system failure related to increased transfusion volume.

## Conclusion

Although investigational use of rFVIIa in trauma patients has shown promising results, the data supporting the use of rFVIIa within trauma have been limited to case series and anecdotal reports. However, in appropriately selected trauma patients rFVIIa may play a role as an adjunctive haemostatic measure in addition to surgical haemostasis.

## Abbreviations

ICU: Intensive Care Unit; rFVIIa: recombinant activated Factor VII; RBC: Red Blood Cells; FFP: Fresh Frozen Plasma; O_2_: Oxygen; MAC: Minimal Alveolar Concentration; ECG: Electrocardiogram; INR: International Normalisation Ratio; PT: Prothrombin Time; aPTT: Partial Prothrombin Time

## Consent

Written informed consent was obtained from the patient for publication of this case report and any accompanying images. A copy of the written consent is available for review by the Editor-in-Chief of this journal.

## Competing interests

The authors declare that they have no competing interests.

## Authors' contributions

KV assisted in the operation contributed to manuscript conception, research, acquisition of data, drafting and writing of the manuscript. FT contributed to manuscript conception, research, acquisition of data, drafting and writing of the manuscript. AP contributed to manuscript conception, research, acquisition of data, drafting and writing of the manuscript. DM contributed to manuscript conception, research, acquisition of data, drafting and writing of the manuscript. SA contributed to manuscript conception, research, acquisition of data, drafting and writing of the manuscript. EL contributed to organizing, drafting and critical review of the manuscript. IGD contributed to organizing, drafting and critical review of the manuscript. NK contributed to organizing and drafting of the manuscript, and critically revised the manuscript. JC contributed to organizing and drafting of the manuscript, and critically revised the manuscript. SZ assisted in the operation and contributed to critical review of the manuscript. AM contributed to manuscript conception, research, acquisition of data, drafting and writing of the manuscript. All authors read and approved the final manuscript.
